# Emerging Role of NLRP3 Inflammasome and Pyroptosis in Liver Transplantation

**DOI:** 10.3390/ijms232214396

**Published:** 2022-11-19

**Authors:** Fernando Lucas-Ruiz, Alejandro Peñín-Franch, José Antonio Pons, Pablo Ramírez, Pablo Pelegrín, Santiago Cuevas, Alberto Baroja-Mazo

**Affiliations:** 1 Molecular Inflammation Group, Biomedical Research Institute of Murcia (IMIB-Pascual Parrilla), University Clinical Hospital Virgen de la Arrixaca, 30120 Murcia, Spain; 2 Hepatology and Liver Transplant Unit, Biomedical Research Institute of Murcia (IMIB-Pascual Parrilla), University Clinical Hospital Virgen de la Arrixaca, 30120 Murcia, Spain; 3 General Surgery and Abdominal Solid Organ Transplantation Unit, Biomedical Research Institute of Murcia (IMIB-Pascual Parrilla), University Clinical Hospital Virgen de la Arrixaca, 30120 Murcia, Spain; 4 Department of Biochemistry and Molecular Biology B and Immunology, Faculty of Medicine, University of Murcia, 30120 Murcia, Spain

**Keywords:** NLRP3 inflammasome, pyroptosis, liver transplantation, ischemia-reperfusion injury, graft rejection

## Abstract

The nucleotide-binding domain leucine-rich repeat-receptor, pyrin domain-containing-3 (NLRP3) inflammasome contributes to the inflammatory response by activating caspase-1, which in turn participates in the maturation of interleukin (IL)-1β and IL-18, which are mainly secreted via pyroptosis. Pyroptosis is a lytic type of cell death that is controlled by caspase-1 processing gasdermin D. The amino-terminal fragment of gasdermin D inserts into the plasma membrane, creating stable pores and enabling the release of several proinflammatory factors. The activation of NLRP3 inflammasome and pyroptosis has been involved in the progression of liver fibrosis and its end-stage cirrhosis, which is among the main etiologies for liver transplantation (LT). Moreover, the NLRP3 inflammasome is involved in ischemia–reperfusion injury and early inflammation and rejection after LT. In this review, we summarize the recent literature addressing the role of the NLRP3 inflammasome and pyroptosis in all stages involved in LT and argue the potential targeting of this pathway as a future therapeutic strategy to improve LT outcomes. Likewise, we also discuss the impact of graft quality influenced by donation after circulatory death and the expected role of machine perfusion technology to modify the injury response related to inflammasome activation.

## 1. Inflammasomes and Pyroptotic Cell Death

**Figure 1:**
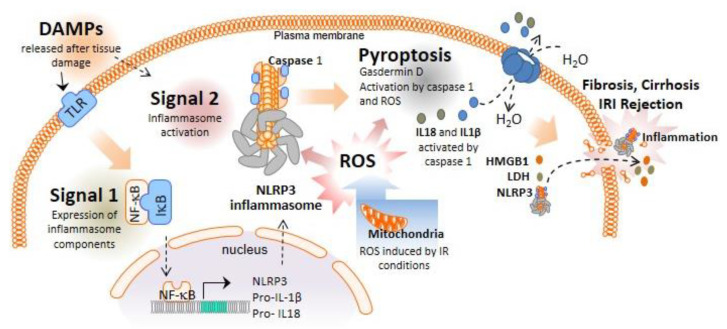
NLRP3 inflammasome and pyroptosis activation during liver inflammation. In the first step of the canonical NLRP3 inflammasome activation, PAMPs and DAMPs stimulate TLR receptors and the translocation of NF-κB to the cell nucleus, which, in turn, increases the transcription of the inflammasome components and expression of pro-IL-1β and pro-IL-18. A second signal, such as P2X7R activation via ATP or crystalline structures, induces the formation of active NLRP3 oligomers leading to the activation of caspase-1. Caspase-1 cleaves the N- terminus of gasdermin D and converts pro-IL-1β and pro-IL-18 into mature IL-1β and IL-18. NLRP3 inflammasome and gasdermin D must be in oxidized form to be active, highlighting the important role of ROS in the regulation of this pathway. Pyroptosis occurs by the insertion of the cleaved gasdermin D N-terminal fragment into the plasma membrane, creating oligomeric pores and allowing the release of proinflammatory mediators such as IL-1β and IL-18 to the extracellular space. Gasdermin D pores also induce cell lysis which further induces inflammation by releasing additional inflammatory intracellular products.

The term “inflammasome” was coined by Tschopp et al. in 2002 to name large multi-protein complexes with high molecular weight that were present in the cytoplasm of stimulated myeloid cells [1]. To date, different inflammasomes have been described, with the nucleotide-binding oligomerization domain and leucine-rich repeat (LRR)-containing receptors (NLRs) being the main family forming inflammasomes; NLRP1, NLRP3, and NLRC4 are the most prominent members of this family. Furthermore, the non-NLR proteins absent in melanoma 2 (AIM2) and pyrin are also capable of forming functional inflammasomes [2]. NLRP3, the most studied inflammasome, forms active oligomers after recognizing damage- or pathogen-associated molecular patterns (DAMPs or PAMPs). These DAMPs and PAMPs can stimulate membrane receptors called Toll-like receptors (TLRs). Stimulated TLRs activate nuclear factor kappa B (NF-κB), which in turn increases the transcription of NLRP3, as well as the expression of proinflammatory cytokines such as prointerleukin (IL)-1β or pro-IL-18 (Figure 1). This is considered the first signal for canonical inflammasome activation. However, a second signal is required to induce the formation of active oligomers of NLRP3, which is mainly mediated by DAMPs, such as crystalline particles, extracellular adenosine triphosphate (ATP) acting through the P2X purinoceptor 7 (P2X7R), or the K^+^ ionophore nigericin (Figure 1).

This activation signal is associated with lysosomal destabilization or mitochondrial dysfunction, inducing cell metabolic changes, reactive oxygen species (ROS) formation, potassium (K^+^) efflux, and calcium (Ca^2+^) influx, which are common signals for NLRP3 activation in response to most of the triggers [3] (Figure 1). The active NLRP3 oligomers recruit the accessory apoptosis-speck-like protein with a caspase activation and recruitment domain (ASC), which oligomerizes in helical filaments that favor caspase-1 activation. The close proximity of pro-caspase-1 zymogens in these ASC filaments within the inflammasome multi-protein complex induces the autoproteolytic activation of caspase-1, initiating the formation of the catalytically active protease caspase-1 (Figure 1). Caspase-1, as the effector enzyme of the inflammasome, proteolytically processes immature proinflammatory cytokines to produce the bioactive forms of IL-1β and IL-18. Additionally, caspase-1 processes gasdermin (GSDM) D protein [4] (Figure 1). This cleavage separates the pore-forming N-terminal domain (GSDMD^NT^) from the gasdermin-C-terminal repressor domain. The C-terminal domain acts as an inhibitor of the N-terminal domain to avoid its pore-forming conformation. Once processed, GSDMD^NT^ binds to the plasma membrane to form pores after homo-oligomerization, releasing mature IL-1β and IL-18 and enabling the entry of water into the cytosol; this induces cellular blebbing and plasma membrane rupture, leading to pyroptotic cell death [5]. Finally, pyroptotic cells burst, with the subsequent release of intracellular components, such as the alarmin high mobility group box 1 (HMGB1), leading to a highly proinflammatory environment [6] (Figure 1).

## 2. Role of the NLRP3 Inflammasome and Pyroptosis in Liver Diseases Necessitating Transplantation

**Figure 2:**
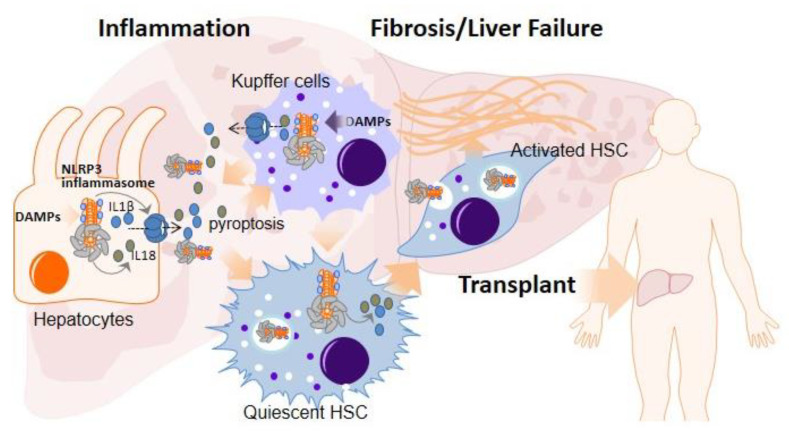
Activation of inflammasome/pyroptosis pathway in liver diseases. Activation of inflammasome in hepatocytes and KCs in early stages of inflammation associated to several liver diseases such as ALD, NAFLD or virus infection induce the release of a plethora of pro-inflammatory compounds, which will active HSCs and evoke an state of fibrosis which can degenerate to the end stage cirrhosis. This process can lead to liver transplantation as ultimate solution.

Liver cirrhosis, a late stage of fibrosis, caused by long-term liver damage, is among the liver diseases that require LT. Inflammation is considered to be the main factor involved in liver damage, and fibrosis in the liver occurs as a compensatory response to liver inflammation and damage. Inflammatory macrophages are increased by the recruitment of circulating monocytes as a consequence of specific chemokines produced by hepatocytes, stellate cells, and resident liver macrophages (termed Kupffer cells (KCs)) following liver damage [7]. This process rapidly expands the amount of macrophages to protect the liver, but it also leads to an excessive inflammatory/reparative response [7]. The NLRP3 inflammasome is considered to be a main pathway for proinflammatory cytokine release in the liver and is strongly involved in the pathogenesis of the liver fibrogenesis [8,9]. Although the NLRP3 inflammasome has been mainly studied in myeloid cells such as macrophages or KCs, it has also been found to be functional in other non-immune cells such as endothelial cells, hepatocytes, or even HSCs (Figure 2).

In the presence of liver injury, some proinflammatory cytokines such as IL-1β and IL-18 are released, mainly by hepatocytes and KCs, activating hepatic stellate cells (HSCs) through the IL-1 receptor, turning into myofibroblasts and releasing a large amount of extracellular matrix, inducing the formation of scar tissue and leading to liver fibrosis [10] (Figure 2). Moreover, the activation of NLRP3 in primary human hepatocytes has been shown to lead to pyroptotic cell death and the release of inflammasome oligomeric particles into the extracellular space, leading to the activation of HSCs by particle internalization [11] (Figure 2). The activation of all these cells has been correlated with liver inflammation, fibrosis, and cell death in several etiologies associated with fibrosis progression, such as alcoholic liver disease (ALD), nonalcoholic fatty liver disease (NAFLD), including nonalcoholic steatohepatitis (NASH) and viral infection [12]. On the other hand, GSDMD, the executioner of pyroptosis, has also been implicated in NASH progression in humans [13]. Moreover, cardiolipin, which functions upstream of NLRP3 inflammasome activation, has been shown to favor NLRP3 activation and the progression of NASH [14], further supporting the role of NLRP3 inflammasome in NASH [15]. The NLRP3 inflammasome not only plays a role in NASH but is also involved in ALD with important roles of NLRP3 and the inflammasome components Caspase-1, GSDMD, ASC, and IL-1β in the progression of this disease [16,17].

Thus, the therapeutic targeting of NLRP3 or the proteins that accompany the formation of the NLRP3 inflammasome (such as ASC or Caspase-1) or even the released pro-inflammatory cytokines and the pore-forming protein GSDMD has garnered increasing consideration of the experts in the field and is an expanding area of research [18]. In this regard, there are multiple pharmacological compounds, including inhibitors/antagonists of DAMPs, P2X7R, IL-1β signaling, caspase-1, NF-κB, and antioxidant molecules that have demonstrated the capacity to dampen the NLRP3 inflammasome and pyroptosis in the liver, showing promising beneficial effects in the prevention of liver disease progression (Table 1). Most of these studies have been carried out in animal models, but some clinical trials have also been conducted in humans. These include an analysis of the effect of the P2X7R inhibitor SGM-1019 in NASH, two trials with the recombinant IL-1 receptor antagonist anakinra in AH, and the utility of the IL-1β blocking antibody canakinumab in AH (Table 1). Nevertheless, liver toxicity has been observed for some of the compounds (Table 1), and further therapy improvement is required to establish these drugs in the clinical setting.

## 3. Role of NLRP3 Inflammasome and Pyroptosis in Ischemia–Reperfusion Injury

Hepatic ischemia–reperfusion (I/R) injury (IRI) is a serious setback that limits the course of LT and can lead to serious postoperative difficulties, including acute rejection [39]. IRI can be divided into two distinct stages; the first stage is ischemia, wherein hepatocytes are deprived of nutrients and oxygen for a prolonged period of time, resulting in massive parenchymal cell death. The second stage is reperfusion, wherein an innate immune response and sterile inflammatory response are initiated. It has been widely described that inflammatory processes occur in IRI, in which the NLRP3 inflammasome plays a crucial role [40]. Reperfusion injury derives mainly from toxic ROS generated during ischemia and is amplified when oxygen is reintroduced into ischemic tissues. Besides ROS, there are several other DAMPs (including ATP, uric acid, cholesterol crystals, DNA fragments, or free fatty acids) that activate the NLRP3 inflammasome and an inflammatory response [40]. These DAMPs are derived from damaged hepatocytes and promote NLRP3 inflammasome activation in KCs and reperfusion-dependent infiltrating macrophages [40]. Moreover, during IRI, NLRP3 inflammasome pathway upregulation leads to pyroptotic cell death via GSDMD cleavage and plasma membrane permeabilization. There are two types of ischemia: warm and cold ischemia. Different studies have focused on unraveling the mechanisms of activation of the NLRP3 inflammasome and testing therapies to block it using warm I/R models [40]. For example, a recent study has shown that fisetin acts as a blocker of NLRP3 activation through the GSK3β/AMPK/NLRP3 pathway [41]. Likewise, pre-treatment with 25-hydroxycholesterol helps activate mitophagy during IRI and reduces the amount of NLRP3, pro-caspase1, caspase1, pro-IL-1β, and IL-1β proteins [42]. Other authors also found that autophagy is essential for a better prognosis of IRI, as the inhibition of the autophagic process leads to the activation of the NLRP3 inflammasome and aggravates IRI [43,44,45]. Docosahexaenoic acid, the main component of omega (ω)-3 polyunsaturated fatty acids, is associated with an upregulation of the PI3K/Akt pathway, preventing oxidative stress in hepatocytes during IRI and reducing the expression of NLRP3 and related cytokines [46]. Moreover, microtubule affinity-regulating kinase 4 (MARK4) inhibition by in vivo RNA interference inhibited IRI-induced NLRP3 inflammasome activation in mice [47]. On the other hand, the genetic upregulation of the canonical and non-canonical pathways of the inflammasome [48] carrying out pyroptotic cell death via GSDMD [48,49] during I/R has also been described.

**Figure 3:**
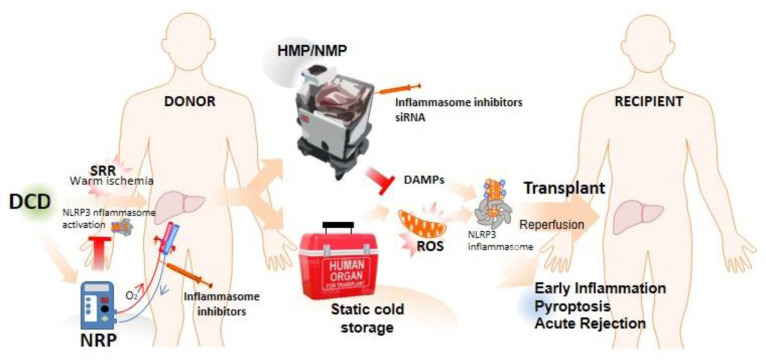
Inflammasome activation during LT. During transplantation surgery, liver IRI increases the rate of acute and chronic rejection and is estimated to be responsible for 10% of early organ failure. DCD has been increasing in recent years, attempting to alleviate the deficit in the number of available organs for transplantation. However, warm ischemia associated with the super-rapid recovery of DCD organs is related to higher NLRP3 activation compared with DBD donors. In this regard, the introduction of the NRP technique has reduced ischemic damage from DCD. Moreover, NRP could be used as the first line of inflammasome inhibitor drug delivery already in the donor. During cold ischemia storage, multiple ROS and DAMPs are released from damaged cells, and these molecules activate the NLRP3 inflammasome and ultimately pyroptosis during reperfusion, which have an impact in the inflammatory response during the early stage of acute rejection in liver transplantation. Recently, the introduction of the extracorporeal machine perfusion technique, HMP and NMP, has improved the liver transplant outcome by NLRP3 inflammasome inhibition during I/R. Furthermore, MP appears as a contrasted platform for the use of specific drugs or even siRNA delivery.

Warm ischemia is mainly observed in vascular occlusion associated with hepatic resection, trauma, or hemorrhagic shock, but it is also found in donation after circulatory death (DCD) during LT. Even though donation after brain death (DBD) accounts for the majority of organ donors, there is an increasing interest in expanding the donor pool [50]. Consequently, DCD has been introduced and is contributing to further increasing the number of donations in numerous countries [51]. Despite continuous improvement, DCD is related to warm ischemia time after circulatory arrest and with further complications when compared to DBD donors [52] (Figure 3). In this regard, it has been demonstrated that NLRP3, IL-1β, and IL-18 protein expression, as well as caspase-1 activity, was higher in hearts from DCD mice compared with DBD after warm ischemia, which was decreased treating donors with an inhibitor of NLRP3 [53]. Moreover, the increased expression of key genes in the TLR4 and inflammasome pathways involved in sterile inflammation has been identified in high-risk donor kidneys [54]. In order to minimize the length of warm ischemia and its deleterious impact, postmortem normothermic regional perfusion (NRP) for DCD is being implanted as an alternative to super-rapid recovery (SRR) [55]. After death declaration in the hospital, femoral vasculature is cannulated to reperfuse and reoxygenate abdominal organs [56]. NRP has been shown to reduce biliary complications, mainly ischemic-type lesions, and to improve DCD liver graft survival in comparison with the SRR technique [55,57] (Figure 3). However, there is no published information that directly relates NRP with a lower release of DAMPs or the activation of the inflammasome.

On the other hand, during LT, a graft undergoes cold ischemia preceding implantation in the recipient [58]. It is well known that the standard static cold-storage protocol causes serious injury to organs, including those considered good quality [59]. In this context, extracorporeal machine perfusion (MP) is emerging into clinical practice to maintain and deliver organs supplied with oxygen and nutrients prior to implantation [60] (Figure 3). Nowadays, basically two variants of machine perfusion are available. On the one hand, sub-normothermic (20–25 °C) and hypothermic (2–10 °C) machine perfusion (SNMP and HMP) require a red-blood-cell-free solution with physically diluted oxygen. On the other hand, normothermic machine perfusion (NMP) emulates physiologic liver perfusion using a warm (35.5–37.5 °C) red-blood-cell-based perfusate, providing a variety of potential benefits [61]. The NLRP3 inflammasome pathway has recently been studied comparing cold-storage conditions and SNMP [62], HMP [29,63], or NMP [64] in ex vivo LT models. SNMP was able to reduce the role of IL-18 and increase the role of the anti-inflammatory mediator IL-1 receptor antagonist (IL-1RA) mediated by a reduced NLRP3 response, compared with cold storage in a porcine LT model [62]. Likewise, cold storage led to a stable pattern of IL-18-induced liver damage, whereas SNMP led to a resolution of the pro-inflammatory response. He et al. [63] revealed that reperfusion injury induces liver damage and activates the NLRP3 inflammasome in a DCD model in rats. The pretreatment of DCD rat livers with HMP inhibited NLRP3 inflammasome activation and attenuated liver IRI. Oxidative stress attenuation as a result of HMP downregulates the NLRP3 inflammasome and, thus, offers superior protection compared to the traditional cold-storage method of organ preservation. Yu et al. [29] treated the perfusate of the HMP system with the specific NLRP3 inflammasome inhibitor MCC950 in a DCD LT pig model. MCC950-treated livers suffered the lightest IRI and functioned better after transplantation. NMP has been associated with a reduced inflammatory response [62,65], and recent clinical trials have suggested that NMP is associated with a less severe IRI, reduced early allograft dysfunction, and less biliary complications when compared to cold storage [60]. However, Scheuermann et al. [64] observed the presence of inflammatory molecules, including DAMPs and cytokines, generated during NMP in a rat model. They reported a time-dependent increase in the plasma levels of HMGB1 and extracellular DNA, beyond TNFα. Moreover, NLRP3 inflammasome priming and cell death activation were more reduced in grafts preserved at SNMR conditions than in those with NMR.

Taken together, this evidence supports the involvement of the NLRP3 inflammasome and downstream pyroptosis in I/R-related damage after LT, suggesting NLRP3 as an attractive novel target to improve LT.

## 4. NLRP3 Inflammasome Activation in the Early Inflammation Stage after LT: Implications in Acute Rejection

Although the survival rate after LT is elevated, liver allograft rejection remains a significant cause of morbidity and graft failure in LT patients. Up to 35% of patients may experience at least one episode of acute rejection, and 2% to 3% will suffer chronic rejection [66]. Rejection after LT is often characterized, among other things, by the infiltration of inflammatory cells. IL-1β expression has been shown to be upregulated during allograft rejection, and recent studies have revealed that inflammasomes are responsible for initiating inflammation in the early stage of acute organ rejection in LT [67]. In a rat model of LT, treatment with the caspase-1 inhibitor Ac-YVAD-CMK significantly diminished the rejection activity index compared with the untreated group, in a magnitude similar to that of isografts [33]. Moreover, *Pycard* and *Il1b* expression in the liver and serum IL-1β levels remained low in comparison with control allografts when animals were treated with Ac-YVAD-CMK. Likewise, Yu et al. evaluated the protective effects of the selective NLRP3 inhibitor MCC950 in a pig model of LT [29]. Their findings indicated that the downregulation of the NLRP3/caspase-1/IL-1β pathway by MCC950 improves transplantation outcome, as measured by the Model of Early Allograft Function (MEAF) score, which has recently been suggested to classify early graft dysfunction after LT [68]. Furthermore, our previous study suggests that extracellular ATP is important as a DAMP in the establishment of allograft rejection during mismatched tissue transplantation through NLRP3 inflammasome activation and IL-18 production [69]. Macrophagal P2X7R, which helps to maintain high extracellular ATP levels in mismatched tissue grafts at early time points after transplantation, functions in an autocrine fashion; it is first activated by extracellular ATP, and the activated form in turn activates the NLRP3 inflammasome, which exacerbates the alloimmune response.

Likewise, recent studies highlight that mitochondrial DNA (mtDNA), considered a potent proinflammatory DAMP, could be released into the circulation as a consequence of liver injury, and has been associated with adverse events after LT [70]. Plasma mtDNA levels were elevated in all patients after LT compared with healthy controls, and 5 days after transplantation, circulant mtDNA come back to normal levels. The patients who developed early allograft dysfunction in the early postoperative period presented elevated mtDNA levels, suggesting a possible strong role for mtDNA release in early allograft dysfunction in LT associated with worse survival [71]. mtDNA have antiviral properties inducing inflammatory processes by means of the activation of TLR9 [72]. Previous studies suggest that TLR9 activates NLRP3 inflammasome via NFkB [73]; however, aditional mechanisms could be involved in this regulation, as a previous publication by our group demonstrated that the increased expression of NLRP3-associated proteins is not enough to induce the NLRP3 inflammasome inflammatory response [74]. Cytosolic DNA detection is a central mechanism for detcting infections, and the DNA effector Stimulator of Interferon Genes (STING) is important for the innate immune system [75] and essential in the inflammatory response induced by cytosolic nucleic acids [76]. Cyclic GMP–AMP synthase (cGAS) is a pathway that induces STING activation. The cGAS–STING axis has a basic role in the activation of the AIM2 inflammasome that is dispensable for DNA-mediated inflammasome activation in human myeloid cells [77]. Instead, cytosolic DNA detection mediated by the cGAS–STING axis induces pyroptosis and NLRP3 activation [77]. It has been suggested that STING activates NLRP3 through a potassium efflux-dependent mechanism [78]. Furthermore, it is known that the NLRP3–inflammasome is a sensor of organelle dysfunction [79] and that cGAS/STING mediate lysosomal cell death. The induction of lysosome integrity destabilization can modify K (+)-permeable channels/transporters in the plasma membrane and regulates NLRP3 [80]. This mechanism could be blocked by the pharmacological inhibition of lysosome function [78]. Likewise, recent studies also show that X-box binding protein 1 (XBP1) regulates STING signaling and the subsequent NLRP3 activation during liver fibrosis, suggesting that macrophage self-mtDNA cytosolic leakage activates cGAS/STING/NLRP3 pathway providing a novel target against liver fibrosis [80]. Thus, the crosstalk between cGAS–STING and the NLRP3 inflammasome appears to have a basic role in the inflammatory response induced by cytoplasmic DNA, and this mechanism could be related to the inflammatory response and the rejection after LT.

All of these studies indicate that the inhibition of the NLRP3 inflammasome may attenuate inflammation during LT rejection. Nevertheless, induction of DAMPs have already been detected in donors [81], including mitochondrial-derived DAMPs, which have been demonstrated to be associated with early allograft dysfunction [82] and even promote allograft rejection through endothelial cell activation [83].

## 5. Conclusions and Future Directions

LT is the ultimate solution for end-stage liver disease. The liver is the second most-commonly transplanted organ after the kidney. In 2020, 8906 patients received an LT, and 11,886 patients were on the waiting list. As previously mentioned, the inflammasome/pyroptosis pathway affects all of the stages involved in LT, including underlying diseases, I/R, and graft rejection. The life expectancy of both grafts and patients has not improved in recent decades; therefore, new approaches to therapy and targets are urgently required. We believe that a reduction of damage evoked during organ recovery and storage is a goal to improve both the quality of marginal livers and the outcome of liver grafts. Although most of the information on NLRP3 inflammasome activation and its implication for LT outcome focuses on the short-term post-LT, further understanding how NLRP3 inflammasome activation is regulated throughout the chronic post-transplant period could be of great importance. Among the different future strategies, the inhibition of the inflammasome may have a prominent place. Pharmacological inhibitors against several steps of the inflammasome activation pathway are currently one of the most significant topics. Since 2016, several small pharmaceutical companies have developed specific NLRP3 inhibitors, and in the past year, large companies have scooped up these smaller companies in huge deals or have started developing inhibitors of their own. Nevertheless, Torreya Capital’s Biopharmaceutical Sector Update Market Outlook ranked “Inflammasome Science” as one of the top five biopharma events of 2020 [84]. Ex vivo MP, beyond the beneficial effect exerted on inflammatory responses, could provide an excellent platform for therapeutic interventions during ex vivo liver preservation [85,86], facilitating the use of specific drugs or even gene silencing with small interference RNA [87,88] (Figure 3). However, the MP technique is relatively new, with only a small number of transplant centers having access to commercially available perfusion machines [89]. On the other hand, NRP, which is a more widely implanted technique for DCD organ recovery [90], could have had a huge incidence on inflammasome activation during warm ischemia, although Hessheimer et al. have recently suggested that cold ischemia time remains an independent predictor for graft loss among DCD livers despite NRP [91]. The combination of NRP to avoid warm ischemia and NMP to overcome cold storage would be an ideal scenario in the future of liver transplantation in relation to the control of inflammasome activation. Nevertheless, NRP might be useful as a first delivery route for drugs designed to inhibit the inflammasome pathway already in the donor (Figure 3).

Furthermore, additional human trials are needed to elucidate convenient pharmacological approaches to avoid liver toxicity effects observed for some of these anti-inflammatory compounds in order to determine the possible use of these drugs to prevent liver inflammation and rejection in transplant patients. Therefore, it is important to determine the mechanisms involved in the toxic effects described in order to clarify whether these effects could be extrapolated to compounds that act on a similar molecular target, or alternatively, are effects associated with other aspects or actions of the studied drugs.

## Figures and Tables

**Table 1:** List of the principal drugs targeting the NLRP3 inflammasome and pyroptosis pathway with protective action in liver pathologies.

Drug	Target	Action	Liver Toxicity	Clinical Trial	References
**Allopurinol**	DAMPs inhibitor	Alcoholic hepatitis. NASH	Minor. Rare acute liver injury		[19,20,21]
**Probenecid**	DAMPs inhibitor	Alcoholic hepatitis	Minor		[19]
**SGM-1019**	P2X7 Antagonist	Liver fibrosis; NASH	n.d.	NCT03676231	[22,23]
**IMD-0354**	NF-κB inhibitor	Liver fibrosis	n.d.		[24]
**Pyrrolidine dithiocarbamate**	NF-κB inhibitor	Liver injury; liver cirrhosis	n.d.		[25,26]
**MCC950**	NLRP3 inhibitor	NASH; liver fibrosis; cholestatic liver injury; rejection	n.d.		[27,28,29,30]
**Anakinra**	IL-1 receptor antagonist	Alcoholic hepatitis	Rare acute liver injury	NCT04072822 NCT01809132	[31]
**Canakinumab**	IL-1β blocking antibody	Liver fibrosis	Rare acute liver injury	NCT03775109	[32]
**Ac-YVAD-CMK**	Caspase-1 inhibitor	Rejection	n.d.		[33]
**Dimethyl fumarate**	Gasdermin inhibitor	ALD	No		[34,35]
**Ac-DMPD/DMLD-CMK**	Gasdermin inhibitor	Acute liver injury	n.d.		[36]
**Ginsenoside-Rg1**	Antioxidant	Acute liver injury	No		[37]
**Naringenin**	Antioxidant	NAFLD	n.d.		[38]

## Data Availability

Not applicable.
